# Pedunculated Natal Tooth: A Case Report

**DOI:** 10.7759/cureus.25992

**Published:** 2022-06-16

**Authors:** Dieter Brummund, Angela Chang, Joseph Michienzi

**Affiliations:** 1 Plastic Surgery, Larkin Community Hospital, Miami, USA; 2 Anesthesiology, Aventura Hospital and Medical Center, Miami, USA; 3 Oral Maxillofacial Surgery, Aventura Hospital and Medical Center, Miami, USA

**Keywords:** riga-fede disease, pedunculate tooth, natal tooth, mandibular tooth, breast feeding, newborn tooth, accessory tooth, neonatal tooth

## Abstract

Natal teeth are teeth present at birth and are a rare finding. They most commonly occur in the mandibular incisor region and are thought to occur as an accelerated premature growth of normal primary teeth. They may present in the varying stages of tooth eruption and rarely on a pedunculated stalk of alveolar mucosa as described in this case. Natal teeth may be surgically extracted if difficulty feeding or ventral tongue ulceration develops. This report presents the case of an unusual pedunculated natal tooth in a newborn at a community hospital and describes its surgical management.

## Introduction

Newborn teeth are rare with a prevalence between 1:1,000 and 1:30,000 depending on the population studied. Traditionally they may be an omen of good or misfortune. Newborn teeth are distinguished by their age of presentation. They are natal teeth if present at birth. They are neonatal teeth if they erupt within the first 30 days of life. Natal teeth occur more frequently than neonatal teeth at a ratio of 3:1. Approximately 6-32% are familial with an autosomal dominant penetrance [1]. They are associated with cleft lip and palate and some congenital craniofacial syndromes including Ellis-van-Creveld syndrome, Hallerman Streiff syndrome, and Jadassohn-Lewandowski syndrome. Natal teeth may also be associated with environmental factors such as infection, fever, endocrine disturbance, superficial position of tooth germs, and exposure to toxins. The pathogenesis of natal teeth was previously thought to be due to aberrant anatomy with a superficial position of teeth germs. However, more recently, natal teeth are thought to be due to an accelerated or premature development pattern of the normal primary deciduous teeth [1].

Natal teeth usually occur in pairs. The majority of the times they are due to the premature eruption of a normal tooth. Natal teeth may be supernumerary 10% of the time, a finding associated with syndrome pathologies and warrants further systemic workup. Natal teeth most commonly occur at the mandibular central incisor region with an incidence of 85%, followed by the maxillary incisor region at 11%, the mandibular canine and molar region at 3%, and finally the maxillary canine and the molar region at 1%. This pattern is thought to coincide with the normal order of eruption for the primary deciduous teeth. Histologically the majority are dysplastic or hypo-mineralized with irregular dentin, osteodentin, and incomplete root formation [2].

Evaluation of a patient with a natal or neonatal tooth begins with a detailed intra-oral examination with special attention paid to whether the tooth is poorly implanted or excessively mobile as this is correlated with a risk of aspiration. The ventral tongue is examined to assess for sublingual ulcerations or granuloma. A nutrition assessment is made to determine if the tooth is interfering with breastfeeding. Finally, plain film radiography is performed to distinguish between an early erupted primary tooth versus a supernumerary tooth [3]. 

## Case presentation

A newborn female was born at 38 weeks and 6 days of gestation, vaginal delivery, birth weight of 7 lbs 8 oz. The mother was 22 years old with two previous children and negative for infectious diseases including Group B Streptococcus, syphilis, hepatitis B, and HIV. Delivery occurred two hours following rupture of membranes and was complicated by a nuchal cord. The Apgar scores were 9 and 9 at 1 minute and 5 minutes, respectively. One mg vitamin K and the hepatitis B vaccination were given intramuscularly after birth.

**Figure 1:**
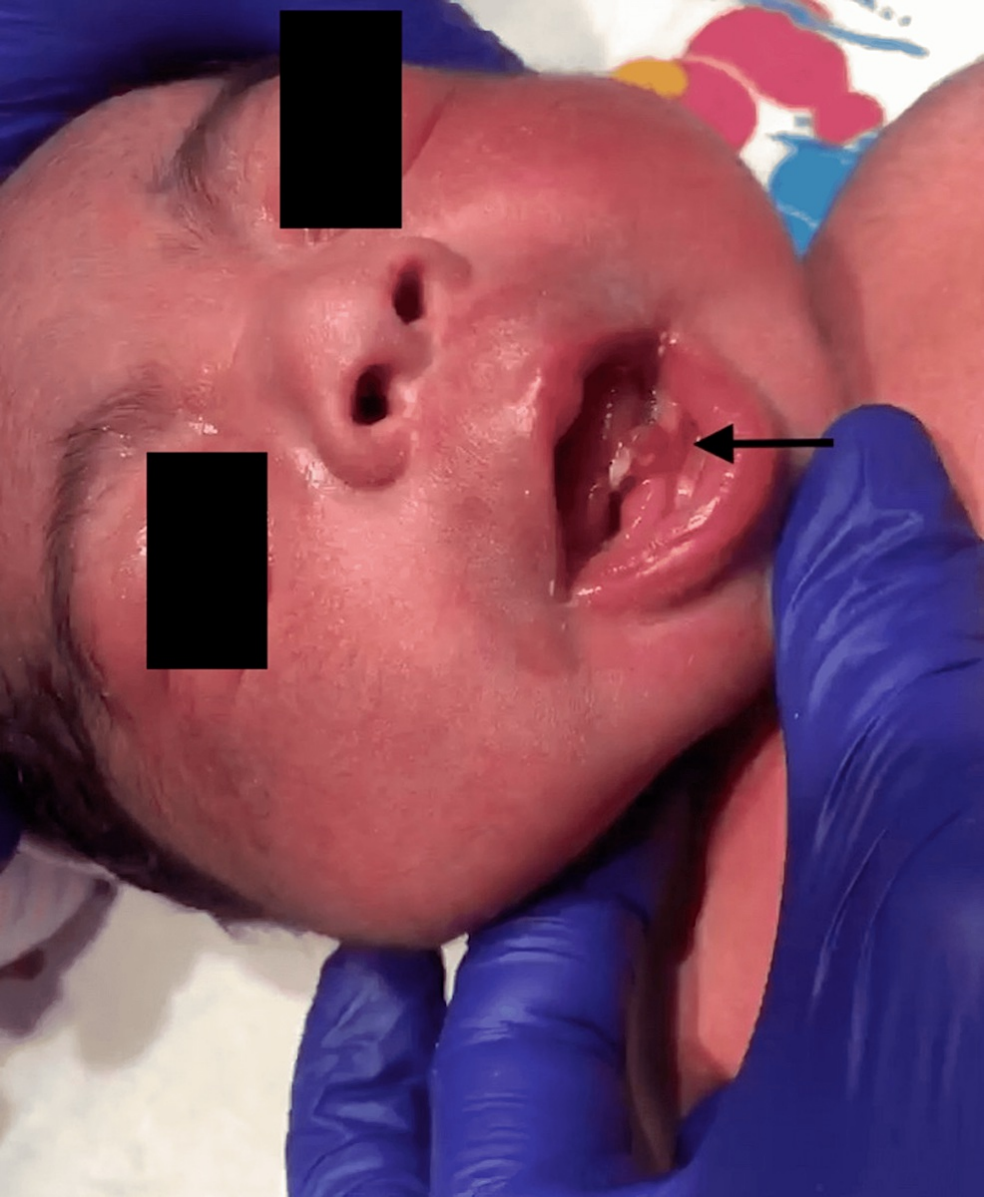
Natal left mandibular incisor Note the oblique incisive edge adjacent to the ventral tongue, an area prone to ulceration, as well as the fleshy pedunculated tooth stalk suggestive of hypoplastic root development.

At delivery, the newborn was found to have a natal tooth at the left mandibular central incisor on a pedunculated stalk (Figure 1). No other teeth were noted. There was no ulceration noted to the ventral surface of the tongue. The tooth was hypermobile on exam with >2 mm mobility (Video 1). Oromaxillofacial surgery was consulted for surgical management given concerns for feeding difficulty.

**Video 1:** Hypermobile natal tooth Note: >2 mm hypermobility on physical exam, which correlates with aspiration risk and is an indication for surgical removal.

The decision was made to proceed with surgical removal. The local gingiva adjacent to the tooth root was anesthetized with 0.1 cc of 1% lidocaine. A 5-0 suture ligature was tied through the base of the root. The natal tooth stalk was sharply excised with scissors. The tooth was sent to pathology, confirming the diagnosis.

The patient tolerated the procedure well. Meticulous oral hygiene with saline rinses was performed following every feeding. The postoperative course was uneventful and the patient was discharged home with her mother the following day.

## Discussion

The differential diagnosis of natal teeth includes both benign and malignant oral pathologies. Natal teeth are most commonly confused with dental lamina cysts, which are white cysts of the alveolar ridge or palate that are transitory in nature, resolving in weeks to months, and attributed to rests of dental lamina and retained keratin. Natal teeth may also be confused with Bohn nodules, an overgrowth of mucus gland tissue on the buccal and lingual aspects of the mandible and maxilla, congenital epulis, sessile or pedunculated growths of gingival tissue, gingival fibroma, hamartoma of the alveolar ridge, and if located in the posterior oral cavity, lymphangioma [3].

The Hebling classification categorizes natal teeth according to their degree of eruption and mobility. Class 1 is a natal tooth with a fully erupted crown that is mobile, poorly fixed to the gingiva, and without a root. Class 2 is a natal tooth with a fully erupted crown that is fixed to the alveolus with a small root. Class 3 is the eruption of only the incisive margin of a natal tooth through the gingiva. Class 4 is an unerupted natal tooth with edema of the overlying gingiva. The natal tooth described in this report would be classified as a Hebling Class 1 and extraction would be indicated based on the aspiration risk given the degree of mobility.

Natal teeth may be left in situ if asymptomatic. Indications for surgical management include a supernumerary tooth, excessive mobility greater than 2 mm, infant tongue ulceration, impairment of breastfeeding, and parental request. A natal tooth could even result in infection and facial abscess [4]. Surgical intervention may involve the extraction of the natal tooth. If hypermobile, supernumerary, or poorly implanted, it is best to extract the tooth. It is important to ensure the patient receives Vitamin K prior to intervention, as it is deficient in the neonatal period and places the patient at an elevated risk of bleeding with any intervention [5].

A report by Sasaki and Matsumine in 2018 reported a similar case of a 1-day-old female with a lower central incisor natal tooth on a pedunculated stalk. A surgical extraction was planned for the following day. However, before this could occur, the patient swallowed the tooth. The tooth was subsequently recovered from the feces for analysis. No image of the gross anatomic specimen was included [6]. This report underscores the importance of prompt surgical extraction of a hypermobile natal tooth to reduce the risk of aspiration or alimentary injury should it spontaneously detach.

## Conclusions

Natal teeth are a rare finding present at birth. Workup includes a detailed physical examination and plain film radiography to distinguish between a prematurely erupted primary deciduous tooth versus a supernumerary tooth, which can be associated with some genetic syndromes. Natal teeth may cause lingual ulceration, difficulty feeding, and parental distress, and are a risk for aspiration if hypermobile. These symptoms warrant surgical extraction of the tooth. Close follow-up with a pediatric dentist is important to closely monitor for subsequent tooth development and other intra-oral pathologies.
